# Chemical Pressure Effect on the Stabilization of Rock-Salt ZnO—Li_n−2_MeO_n−1_ Solid Solutions Synthesized at High Pressure

**DOI:** 10.3390/ma16155336

**Published:** 2023-07-29

**Authors:** Petr S. Sokolov, Andrey N. Baranov, Vladimir L. Solozhenko

**Affiliations:** 1 LSPM–CNRS, Université Sorbonne Paris Nord, 93430 Villetaneuse, France; 2 Chemistry Department, Moscow State University, 119991 Moscow, Russia

**Keywords:** ZnO solid solutions, high-pressure synthesis, chemical pressure, thermal stability, thermal expansion

## Abstract

Metastable ZnO—Li_n−2_MeO_n−1_ (Me = Sc^3+^, Ti^4+^, Ta^5+^) solid solutions with a rock-salt structure were synthesized through the solid-state reaction of ZnO with Li_n−2_Me^n+^O_n−1_ (n = 3, 4, 5) complex oxides at 7.7 GPa and 1300–1500 K. In all investigated systems, single-phase rock-salt solid solutions can be quenched down to ambient conditions in a wide (up to 80 mol% ZnO) concentration range. The phase composition, thermal stability, and thermal expansion of the recovered rock-salt solid solutions were studied by synchrotron powder X-ray diffraction. At ambient pressure, these solid solutions exhibit high thermal stability (up to 1000 K), with the decomposition temperature and decomposition products depending on the nature of the multiple charge cations.

## 1. Introduction

There are a number of crystal structures that have the potential to combine a wide variety of cations in a single compound over a wide range of concentrations. This is necessary in the design of catalytic materials or to control the properties of semiconductors and other functional materials. Examples of such universal structures are perovskites, garnets, spinels, etc. [1,2]. Also, as a universal platform for creating inorganic materials, we can consider the rock-salt structure, which is typical for alkali metal halides or binary ionic compounds such as Me^2+^O oxides. Rock-salt modification is also known for a semiconducting material such as zinc oxide. In this regard, zinc oxide and ZnO-based solid solutions are very promising objects [3,4]. Zinc oxide belongs to the family of wide band gap semiconductors with strong ionic character of chemical bonds [3,4]. Under ambient conditions, ZnO has a hexagonal wurtzite structure (*P*6_3_*mc*, S.G. 186, *w*-ZnO), which changes to a cubic rock-salt structure (*Fm*-3*m*, S.G. 225, *rs*-ZnO) at pressures above 5 GPa [5,6,7,8,9].

The rock-salt structure, in contrast to the wurtzite one, allows the synthesis of ZnO solid solutions with a wide variety of cations. Previously, for Zn^2+^ ← Me^2+^ substitution, isovalent cations (Me^2+^ = Mg^2+^, Ni^2+^, Fe^2+^, Co^2+^, and Mn^2+^) with a tendency to octahedral environments were selected, which significantly affected the thermal stability of the formed solid solutions [8,9,10,11,12,13,14]. The most stable solid solutions with high ZnO content (up to 80 mol%) were those with magnesium and nickel oxides. For example, the cubic Ni_0.2_Zn_0.8_O is stable up to 970 K, whereas the cubic Co_0.3_Zn_0.7_O is stable only up to 670 K. The decomposition products are usually mixtures of two solid solutions, one with a cubic structure and another with a wurtzite structure [8,9,10].

In addition to binary solid solutions, ternary ZnO solid solutions were also synthesized by replacing two zinc cations with a combination of a single-charged lithium cation and a triple-charged metal cation, i.e., 2Zn^2+^ ← Li^+^ + Me^3+^. Extensive solid solutions have been reported for LiFeO_2_ [9,15], LiCrO_2_ [16] and LiTiO_2_ [9,15,17]. Limited cubic solid solutions in the ZnO–Li_2_TiO_3_ system have been described at ambient pressure [18]. As in the case of isovalent substitution, the concentration range and thermal stability of the synthesized solid solutions with the combination of Li^+^ + Me^3+^ essentially depend on the nature of the trivalent cation. Rock-salt (LiTiO_2_)_0.2_(ZnO)_0.8_ is stable up to 800 K, while (LiFeO_2_)_0.2_(ZnO)_0.8_ is stable only up to 670 K [15]. In fact, other examples of ternary cubic ZnO-based solid solutions are not found in the literature.

In the present work, in addition to the system with the trivalent cation scandium, a similar principle has been extended to the four- (3 Zn^2+^ ← 2 Li^+^ + Me^4+^) and five-valent (4 Zn^2+^ ← 3 Li^+^ + Me^5+^) cations, using titanium and tantalum as examples. All selected multivalent cations in the maximum oxidation state (Me^n+^) form individual phases with lithium (Li_n−2_Me^n+^O_n−1_) although not in a rock-salt crystal lattice.

## 2. Experiment

The precursors listed in Table 1 were prepared by solid-state reactions of lithium carbonate (Li_2_CO_3_, Merck, min. 99%) with the corresponding metal oxide (reactive grade; a full list of the starting reagents is given in the Supplementary Materials). Due to lithium evaporation at high temperatures, Li_2_CO_3_ was taken in 5% excess. The fine powder mixtures were pressed into pellets and annealed in an alumina crucible in air at 970 K for 2 h. The product was reground and annealed again at 1070 K for 2 h. According to X-ray diffraction measurements (MZIII Seifert CuKα radiation), all of the as-synthesized precursors were single phase (see Figures S1 and S2 in the Supplementary Materials).

Mixtures of complex oxide and *w*-ZnO (Alfa Aesar, 99.99%) with a ZnO molar fraction (*x*) from 0.5 to 0.8 (with a 0.1 step) were thoroughly ground in an agate mortar with acetone, pressed into pellets and placed in gold capsules. High-pressure synthesis was performed in a toroid-type apparatus at the LSPM–CNRS. The experimental details and pressure–temperature calibration have been described elsewhere [8]. In general, the samples were gradually compressed up to 7.7 GPa, heated for 5 min at the desired temperature (1300–1500 K), then quenched by turning off the power supply and slowly decompressed. After disassembling the high-pressure cell, samples with high ZnO content (*x* > 0.7) usually crumbled into a white microcrystalline powder, while the recovered samples with lower ZnO content retained the tablet shape and looked like dense and well-sintered ceramics. The morphology of the recovered samples was studied by scanning electron microscopy Leo Supra 50VP (Carl Zeiss, Germany).

The high-pressure (up to 5 GPa)—high-temperature (up to 1400 K) behavior of the initial LiScO_2_ complex oxide was studied in situ by synchrotron energy-dispersive X-ray diffraction using the MAX80 multi-anvil system at F2.1 beamline of the DORIS III storage ring (HASYLAB-DESY); the details have been described elsewhere [13,14].

Thermal stability in air at temperatures up to 1200 K was studied by simultaneous thermogravimetric/differential thermal analysis using a Perkin Elmer Diamond Thermal Analyzer (TG/DTA) at a heating rate of 10 K/min.

In situ high-temperature (up to 1100 K) powder X-ray diffraction measurements at ambient pressure were performed at the B2 beamline of the DORIS III storage ring (HASYLAB-DESY) [33]. Debye–Scherrer geometry with a rotating fused silica capillary was used. Diffraction patterns were collected in the 2–70° 2θ-range for 2 min in real time using an OBI image plate detector [34]. A NIST powder LaB_6_ (*Pm*-3*m*, *a* = 4.15695 Å) was used as the standard sample for detector calibration. The furnace temperature was kept constant within 1 K using a Eurotherm temperature controller and Pt10%Rh–Pt thermocouple. The temperature step was 50 or 100 K. Before each data acquisition step, the sample temperature was stabilized for 2–3 min; the acquisition time was 2 min. The collected powder diffraction patterns were analyzed by the Le Bail method [35] using Powder Cell 2.4 [36] and DatLab 1.34 [37] software.

## 3. Results and Discussion

### 3.1. ZnO—LiScO_2_ System

The as-synthesized tetragonal (*I*4_1_/*amd*) LiScO_2_ contained no impurities and had lattice parameters of *a* = 4.187(5) Å and *c* = 9.315(3) Å, which were in good agreement with the literature data [19,20]. It should be noted that the *I*4_1_/*amd* structure is the only crystal form of LiScO_2_ that has been reported. This is a cation-ordered NaCl-like structure. The partial cation-ordering results in the primitive tetragonal body-centered unit cell of LiScO_2_, which can be described as a NaCl superstructure doubled along the *c*-axis. The coordination polyhedron for both lithium and scandium cations is a distorted octahedron, slightly elongated in the *c*-direction, making this structure similar to anatase [19,20].

In a special series of experiments at 5 GPa, the crystal structure of LiScO_2_ was studied in situ up to 1400 K using a MAX80 multi-anvil press and energy-dispersive X-ray diffraction. No melting or phase transition(s) of the pristine *I*4_1_/*amd* tetragonal structure was observed in the investigated temperature range. The LiScO_2_ quenched from 7.7 GPa after a 5-min hold at 1370 K had the initial *I*4_1_/*amd* structure with a small amount of cubic (*Ia*-3) Sc_2_O_3_ impurity; no other cubic phase(s) was observed (see Figure S1a).

**Figure 1:**
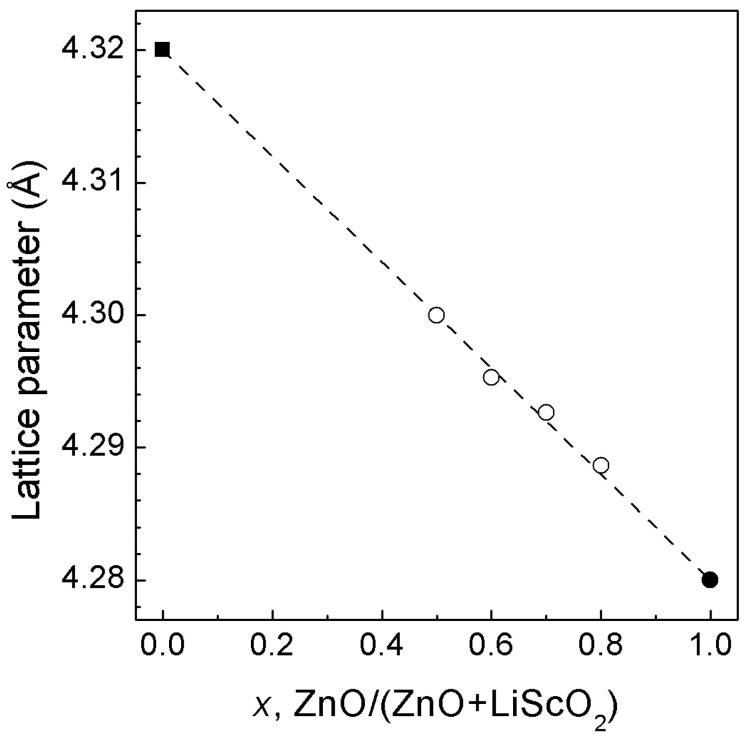
Lattice parameters of *rs*-(LiScO_2_)_1−*x*_(ZnO)*_x_* solid solutions as a function of composition under ambient conditions. The error bars are smaller than the symbols. The dashed line is a guide for the eye. The open circles are our data, and the solid circle is the literature value [7,8,9]. The solid square is our estimate of the lattice parameter of the hypothetical rock-salt LiScO_2_, derived from the simple relationship *a*^3^ = V_tet_/2, where V_tet_ is the unit cell volume of the tetragonal (*I*4*_1_/amd*) phase.

**Figure 2:**
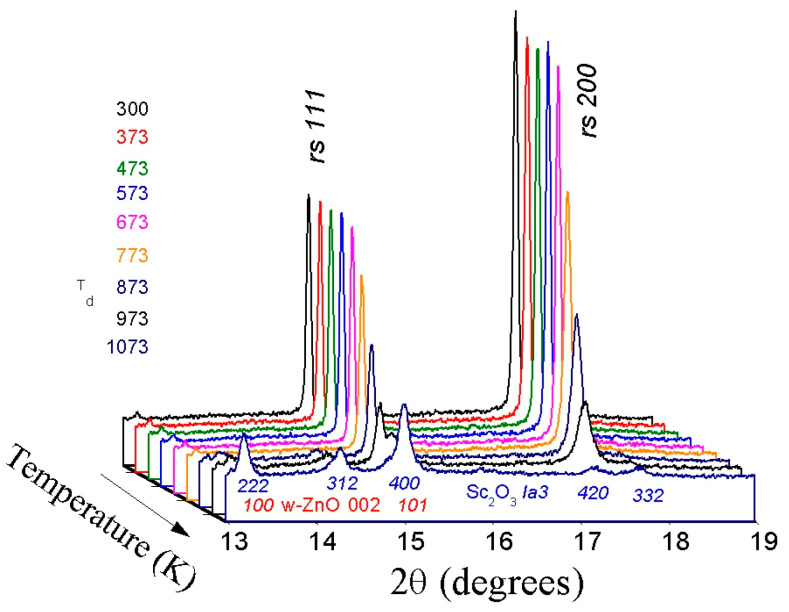
Sequence of synchrotron powder X-ray diffraction patterns of (LiScO_2_)_0.3_(ZnO)_0.7_ solid solution taken during stepwise heating to 1100 K (wavelength λ = 0.65148 Å). The initial rock-salt phase is stable up to 870–900 K; the decomposition products are a mixture of two phases—wurtzite-like (*P*6_3_*mc*) and cubic (*Ia-3*) Sc_2_O_3_.

**Figure 3:**
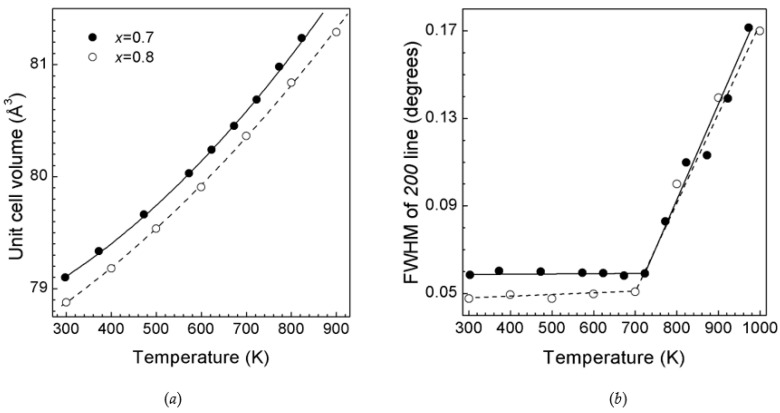
(**a**) Unit cell volumes of *rs*-(LiScO_2_)_1−*x*_(ZnO)*_x_* solid solutions versus temperature at ambient pressure. The solid and dashed lines are the least-squares fits (see Table 2). (**b**) FWHM of the *200* line of *rs*-(LiScO_2_)_1−*x*_(ZnO)*_x_* solid solutions versus temperature at ambient pressure. The solid and dashed lines are guides for the eye. The solid circles correspond to *rs*-(LiScO_2_)_0.3_(ZnO)_0.7_, and the open circles correspond to *rs*-(LiScO_2_)_0.2_(ZnO)_0.8_. In all cases, the error bars are smaller than the symbols.

Four compositions with *x* varying from 0.8 to 0.5 (in steps of 0.1) were synthesized in the ZnO—LiScO_2_ system. The results are summarized in Figure 1, Figure 2 and Figure 3 and Table 2, where the observed ranges of solid solutions are given in terms of *x*, the fraction of Zn^2+^ ions substituted by other cations. In all cases, the recovered samples were white insulating bulks. According to the SEM data, the synthesized solid solutions were dense, practically poreless ceramics with a grain size of about 10 microns.

Figure S3a–c shows the representative X-ray diffraction patterns of the synthesized rock-salt solid solution containing a small amount of Sc_2_O_3_ as an impurity. The sharp symmetrical narrow lines indicate that the solid solution is well crystallized. The dependence of the lattice parameters on the composition of the ZnO—LiScO_2_ system is shown in Figure 1. This dependence is nearly linear and follows Vegard’s law, as in the case of the related ZnO—LiTiO_2_ and ZnO—LiFeO_2_ systems [15].

A sequence of powder X-ray diffraction patterns for the *rs*-(LiScO_2_)_1−*x*_(ZnO)*_x_* solid solution composition with high ZnO content (*x* = 0.7) collected during stepwise heating up to 1100 K is shown in Figure 2. The first signs of change become visible at 873 K, when weak and broad reflections of the wurtzite phase appear, indicating the beginning of the decomposition of the initial single-phase cubic solid solution into a mixture of wurtzite solid solutions and Sc_2_O_3_ according to the following scheme:

*rs*-(LiScO_2_)_1−*x*_(ZnO)*_x_* → *w*-ZnO:Li + Sc_2_O_3_ (1)

where *w*-ZnO:Li is ZnO-Li_2_O solid solutions of different compositions.

The intensities of the lines corresponding to the cubic phase gradually decrease, and these lines disappear completely only at the highest temperatures reached in the experiment (~1100 K). At the same time, according to the simultaneous TG/DTA analysis, the weight loss and any thermal effects are not observed over the whole temperature range studied.

The temperature dependencies of the unit cell volumes of *rs*-(LiScO_2_)_1−*x*_(ZnO)*_x_* solid solutions are shown in Figure 3a. The experimental thermal expansion data were fitted to the following equation:

V(T) = V_0_[1 + α_1_(T − 98) + α_2_(T − 298)^2^] (2)

the coefficients of which are given in Table 2. The unit for V(T) and V_0_ is Å^3^; the units for α_1_ and α_2_ are K^−1^ and K^−2^, respectively. The quadratic dependence of V(T) has been previously observed for ZnO-rich cubic solid solutions in other Li- containing systems [9,10,15], and α_1_ and α_2_ are close to the previously reported values [15].

The temperature dependencies of full width at half maximum (FWHM) for the *200* line (100% intensity) of *rs*-(LiScO_2_)_1−*x*_(ZnO)*_x_* solid solutions are shown in Figure 3b. In comparison, the most intense line *110* of the NIST standard LaB_6_ also has a similar (~0.05°) FWHM, indicating a high degree of crystallinity of the synthesized solid solutions. As the temperature increases, the FWHM of the *200* line of the cubic phase remains almost constant up to 700 K (Figure 3b). Then a sharp (2 to 3 times) increase in FWHM is observed, which begins 100–200 K below the temperature when the decomposition of the cubic phase becomes visible (i.e., the appearance of *w*-ZnO lines). Such FWHM behavior is associated with the segregation of microcrystalline nonequilibrium cubic solid solutions at the nanoscale with the formation of ZnO-enriched and -depleted solid solutions (pre-phase solid solution separation). Then the wurtzite zinc oxide and cubic scandium oxide phases crystallize from these intermediate solid solutions.

Thus, (LiScO_2_)_1−*x*_(ZnO)*_x_* cubic solid solutions of the composition 0.5 ≤ *x* ≤ 0.8 with high thermal stability at ambient pressure were synthesized for the first time at high pressures and high temperatures.

### 3.2. ZnO—Li_2_TiO_3_ System

The as-synthesized monoclinic (*C*2/*c*) Li_2_TiO_3_ contained no impurities (Figure S2) and had lattice parameters of *a* = 5.049(2) Å; *b* = 8.807(1) Å; and *c* = 9.715(6) Å, which were in good agreement with the literature data [22,23,24,25]. This compound is the only precursor known to have a high-temperature phase with a rock-salt structure (*Fm*-3*m*), in which Li^+^ and Ti^4+^ cations are completely disordered in their sublattice. At ambient pressure, the cubic phase has its stability field in the phase diagram at temperatures of 1430–1820 K, i.e., up to the melting point [21,22,23], and can be quenched under normal conditions. Another feature of Li_2_TiO_3_ is that the Ti^4+^ ion has the minimum size (0.60 Å) for the whole studied series of Me^n+^ cations [8,9,12,15].

**Figure 4:**
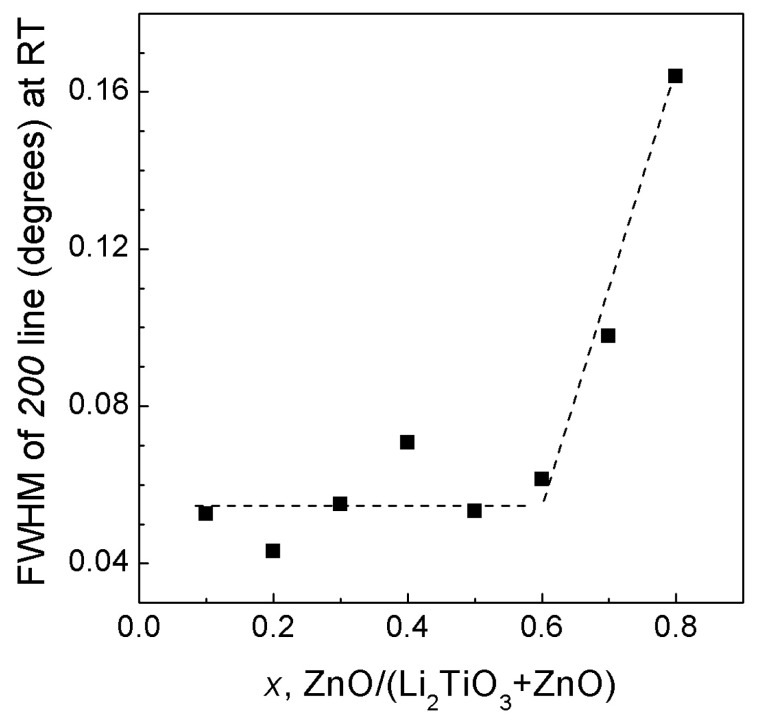
FWHM of the *200* line of the *rs*-(Li_2_TiO_3_)_1−*x*_(ZnO)*_x_* solid solutions versus the composition under ambient conditions (solid squares). The error bars are smaller than the symbols. The dashed lines are guides for the eye.

Eight single-phase cubic solid solutions with *x* varying from 0.8 to 0.1 (in steps of 0.1) were synthesized in the ZnO—Li_2_TiO_3_ system. The powder X-ray diffraction pattern of the solid solution with the maximum ZnO content, (Li_2_TiO_3_)_0.2_(ZnO)_0.8_, is shown in Figure S3d. The diffraction lines of all synthesized solid solutions are perfectly symmetric, the lines themselves are of high intensity; the FWHM of the *200* line depends on the composition as shown in Figure 4. The most ZnO-rich compositions (*x* = 0.8–0.7) have rather broad lines, whereas for compositions with *x* ≤ 0.6, the *200* line width remains almost constant and is equal to ~0.06° (which is close to the line width of the LaB_6_ standard), indicating their high crystallinity.

**Figure 5:**
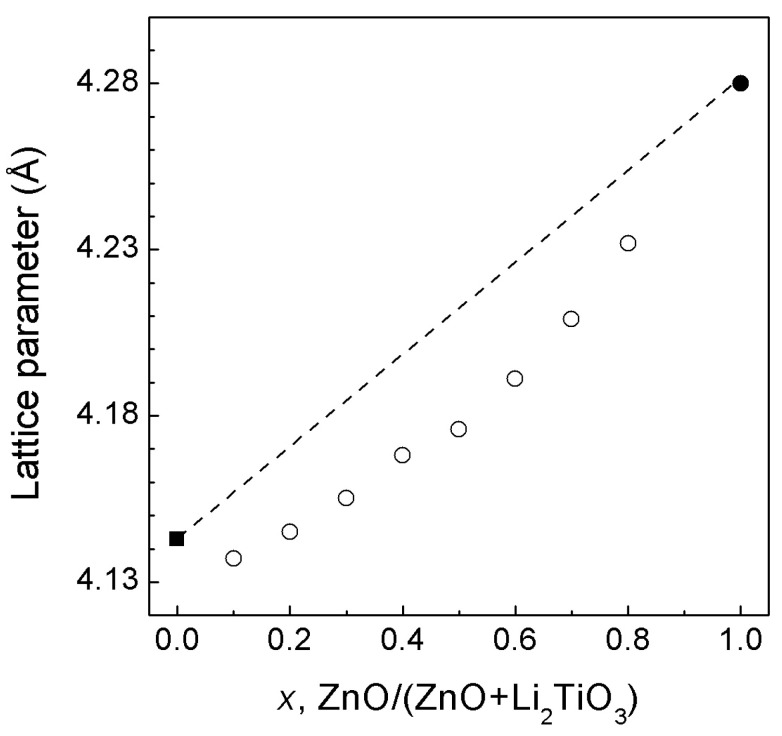
Lattice parameters of *rs*-(Li_2_TiO_3_)_1−*x*_(ZnO)*_x_* solid solutions as a function of composition under ambient conditions. The open circles are our data, and the solid circle [7,8,9] and solid square [26,27] are the literature data. The dashed line represents Vegard’s law.

At room temperature, the dependence of the cell parameter of cubic solid solutions on the composition is nonlinear (Figure 5), with a negative deviation from Vegard’s law. This is indicative of the manifestation of the chemical pressure effect, when the multi-charged cation with a smaller radius produces an electrostatic lattice compression effect [38]. Since in this case, we know the endpoints of the concentration dependence of the *a* parameter, it is possible to quantify this effect from the difference in the lattice parameters and compressibility of *rs*-ZnO [39]. The chemical pressure calculated in this way was 3–5 GPa, depending on the composition of the solid solution. It should be noted that for the ZnO—LiScO_2_ system, such an effect is not observed due to the larger radius of the Sc^3+^ cation compared to Zn^2+^, and a smaller charge compared to Ti^4+^.

It was previously reported that Li_2_TiO_3_ and LiFeO_2_ form continuous NaCl-type solid solutions with MgO at ambient pressure above 1300 K, and a deviation from Vegard’s law was observed [18,40,41,42]. Due to the similarity of the ionic radii and chemical nature of the Zn^2+^ and Mg^2+^ cations, the related effects of incipient immiscibility and/or short-range ordering of cations [41] can occur in ZnO-based cubic solid solutions.

The mechanism of decomposition can be described by the following scheme:

*rs*-(Li_2_TiO_3_)_1−*x*_(ZnO)*_x_* → *w*-ZnO + *rs*-(Li_2_TiO_3_)_1−*y*_(ZnO)*_y_* (3)

**Figure 6:**
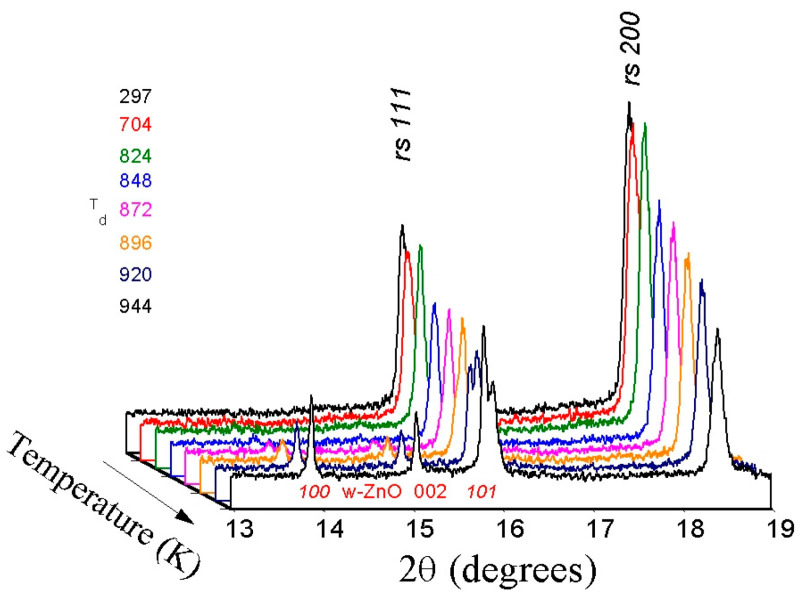
Sequence of synchrotron powder X-ray diffraction patterns of (Li_2_TiO_3_)_0.2_(ZnO)_0.8_ solid solution taken during stepwise heating to 1100 K (wavelength λ = 0.6841 Å). The initial rock-salt phase is stable up to 800–1000 K; the decomposition products are mainly a mixture of two phases—wurtzite and rock-salt.

As the ZnO fraction in the cubic solid solution decreases, the onset temperature of decomposition (T_d_) increases from 870 K for *x* = 0.8 (Figure 6) to 1040 K for *x* = 0.5. Compositions with lower zinc oxide content are kinetically stable up to 1100 K, the maximum temperature available in our experiments. In some cases, small amounts of the *C*2/*c* monoclinic phase, characteristic of Li_2_TiO_3_ in this temperature range, have been observed in the X-ray diffraction patterns of decomposition products [25]. The FWHM values of the diffraction lines of all cubic solid solutions studied remain almost constant with increasing temperature.

**Figure 7:**
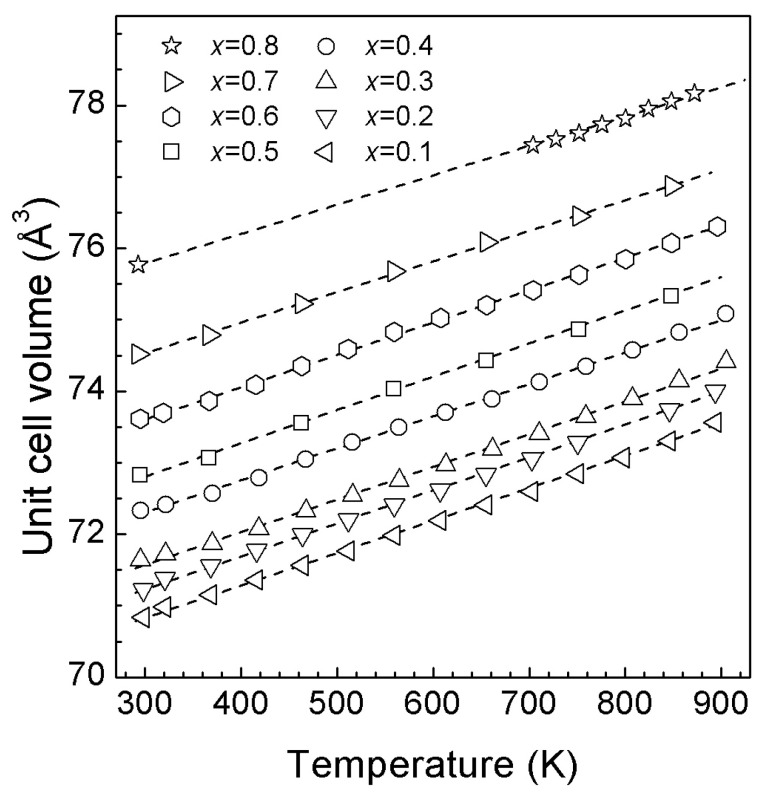
Unit cell volumes of *rs*-(Li_2_TiO_3_)_1−*x*_(ZnO)*_x_* solid solutions versus temperature at ambient pressure. The error bars are smaller than the symbols. The dashed lines are the least-squares fits (see Table 3).

The temperature dependencies of the unit cell volumes of *rs*-(Li_2_TiO_3_)_1−*x*_(ZnO)*_x_* solid solutions are shown in Figure 7.

The experimental thermal expansion data were fitted to the following equation

V(T) = V_0_[1 + α(T − 298)] (4)

where the coefficients of which are given in Table 3. The unit of V(T) and V_0_ is Å^3^, and the unit of α is K^−1^. For these solid solutions, V(T) is linear or quasi-linear. For *x* < 0.6, the dependence of the thermal expansion coefficient on the composition is practically absent (Table 3, Figure S4), whereas for *x* ≥ 0.6, a tendency for its decrease with increasing zinc oxide content is observed. Note that extrapolation to 100% ZnO content (*rs*-ZnO) gives a value close to that previously reported by us [10].

Thus, for the first time, we have synthesized cubic ZnO—Li_2_TiO_3_ solid solutions in a wide concentration range (0.1 ≤ *x* ≤ 0.8), which are characterized by high thermal stability.

### 3.3. ZnO—Li_3_TaO_4_ System

The initial Li_3_TaO_4_ was metastable and had a cubic structure (*Fm*-3*m*) with lattice parameter *a* = 4.215(1) Å, which is in agreement with the literature data (*a* = 4.2207(4) Å [31] and *a* = 4.214(5) Å [32]). Quenched from 7.7 GPa and 1370 K, Li_3_TaO_4_ lost its cubic structure; the X-ray diffraction patterns before and after the high-pressure—high-temperature treatment are shown in Figure S1b.

**Figure 8:**
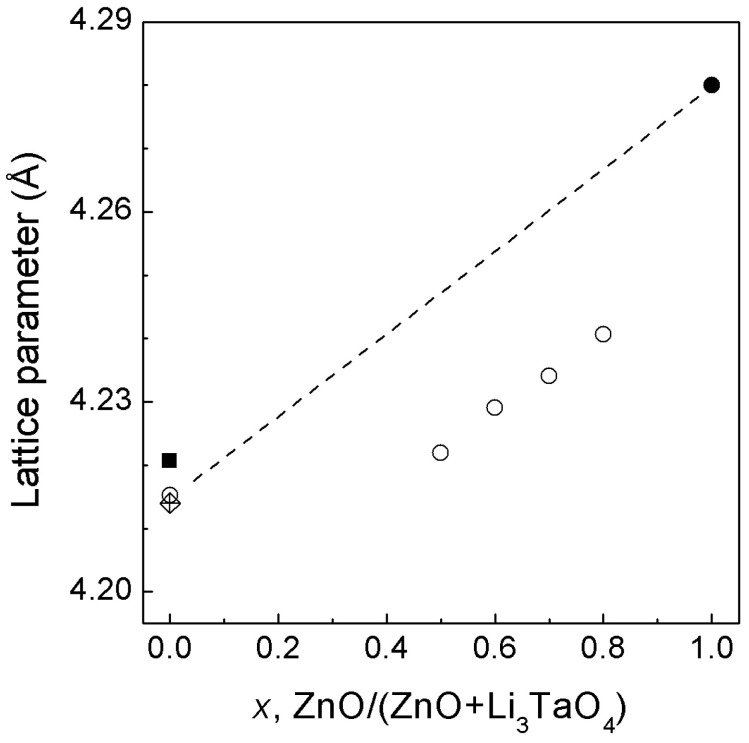
Lattice parameters of *rs*-(Li_3_TaO_4_)_1−*x*_(ZnO)*_x_* solid solutions as a function of composition under ambient conditions. Open circles are our data, and the solid circle [7,8,9], solid square [31], and diamond [32] are the literature data. The dashed line represents Vegard’s law.

**Figure 9:**
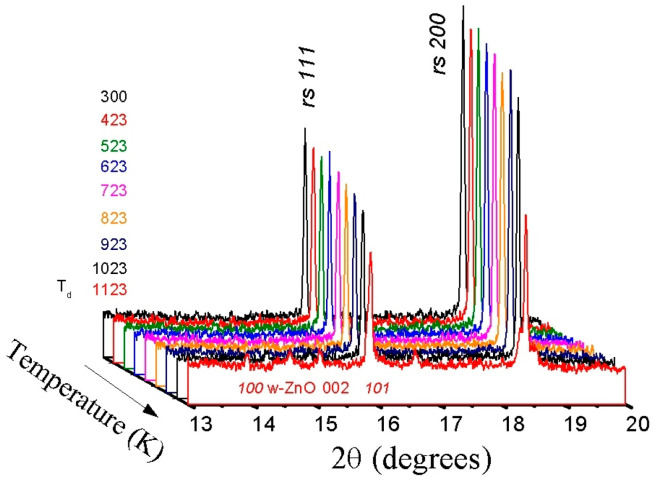
Sequence of synchrotron powder X-ray diffraction patterns of (Li_3_TaO_4_)_0.5_(ZnO)_0.5_ solid solution taken during stepwise heating to 1100 K (wavelength λ = 0.68805 Å). The initial rock-salt phase is stable up to 1000 K; the decomposition products are mainly a mixture of two phases—wurtzite and rock-salt.

**Figure 10:**
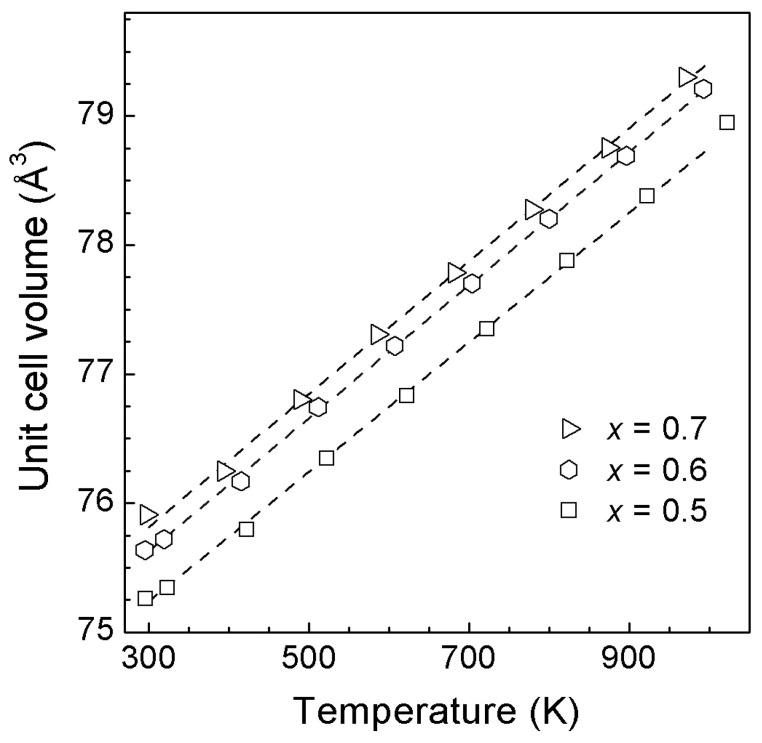
Unit cell volumes of *rs*-(Li_3_TaO_4_)_1−*x*_(ZnO)*_x_* solid solutions versus temperature at ambient pressure. The error bars are smaller than the symbols. The dashed lines are the least-squares fits (see Table 4).

Seven compositions with *x* varying from 0.8 to 0.3 (in steps of 0.1) were synthesized in the ZnO—Li_3_TaO_4_ system. The results are summarized in Figure 8, Figure 9 and Figure 10 and Figure S5 and Table 4, where the observed ranges of solid solutions are given in terms of *x*, the fraction of Zn^2+^ ions substituted by other cations. In all cases, the recovered samples were white insulating bulks.

The powder X-ray diffraction pattern of the (Li_3_TaO_4_)_0.5_(ZnO)_0.5_ solid solution is shown in Figure S3f, which indicates that it is cubic and single phase. The profiles of the diffraction lines are perfectly symmetric, and the lines themselves are of high intensity. Note that in the related system, MgO-Li_3_TaO_4_ solid solutions with a cubic structure do not form [43].

The diffraction pattern of the solid solution with nominal composition (Li_3_TaO_4_)_0.2_(ZnO)_0.8_ shows a more complex picture (Figure S3e): two groups of narrow and intense lines are seen, both of which can be described as structures belonging to the *Fm*-3*m* space group. The lattice parameter of the first (18 vol% content) is 4.278(2) Å, and the lattice parameter of the second is 4.2404(1) Å (82 vol% content) while other lines are absent. The presence of two cubic NaCl-like structures with slightly different lattice parameters may indicate the presence of micron-scale regions with different compositions. With the increasing temperature, the first group of lines gradually decreases in intensity and disappears completely at 620 K. The intensity of the second group of lines also decreases smoothly up to 1070 K. Previously, only *rs*-Ni_1−*x*_Zn*_x_*O cubic solid solutions [10] have shown such high thermal stability.

For the initial composition (Li_3_TaO_4_)_0.7_(ZnO)_0.3_, the formation of a mixture of phases is observed, among which we can distinguish rock-salt and wurtzite phases. Also observed is a group of lines that cannot be described by any known structure but that are similar to the lines of the *rs*-Li_3_TaO_4_ decomposition products.

A negative deviation from Vegard’s law is observed for the dependence of the cell parameter of cubic solid solution on the composition (Figure 8). The calculated values of chemical pressure vary in the range of 3–5 GPa, depending on the composition of the solid solution.

For the compositions (Li_3_TaO_4_)_0.3_(ZnO)_0.7_, (Li_3_TaO_4_)_0.4_ (ZnO)_0.6,_ and (Li_3_TaO_4_)_0.5_(ZnO)_0.5_, the cubic structure is stable up to about 1000 K (Figure 9). At ~1100 K, numerous new lines of low intensity appear, which we could not interpret.

The mechanism of decomposition of cubic solid solutions in this case is as follows:

*rs*-(Li_3_TaO_4_)_1−*x*_(ZnO)*_x_* → *w*-ZnO + *rs*-(Li_3_TaO_4_)_1−*y*_(ZnO)*_y_* + phase(s) X (5)

The temperature dependencies of the unit cell volumes of *rs*-(Li_3_TaO_4_)_1−*x*_(ZnO)*_x_* solid solutions are shown in Figure 10. The experimental thermal expansion data were fitted to Equation (4), the coefficients of which are given in Table 4.

Thus, we have demonstrated for the first time that synthesis at high pressure and high temperature allows for single-phase cubic solid solutions in the ZnO—Li_3_TaO_4_ system to be obtained in a relatively narrow concentration range (0.5 ≤ *x* ≤ 0.8). The synthesized solid solutions exhibit high thermal and phase stability.

### 3.4. General Remarks

**Figure 11:**
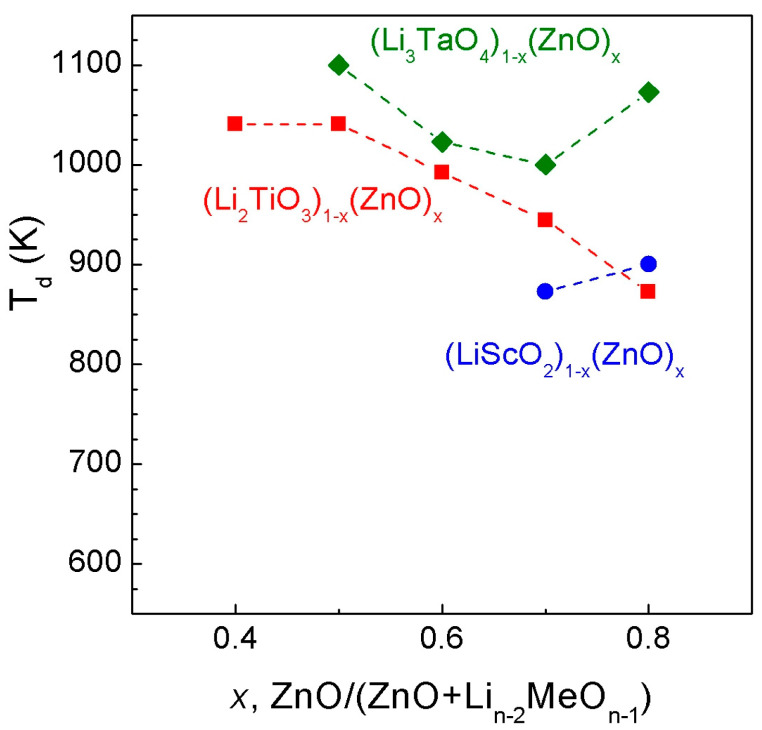
Comparison of the phase stability of rock-salt ZnO-Li_n−1_MeO_n−2_ (Me^n+^ = Sc^3+^, Ti^4+^, Ta^5+^) solid solutions at ambient pressure. Below the dotted lines are the kinetic stability fields of the corresponding cubic phases; above, the decomposition with formation of different phases is observed, n = 3, 4, 5 is an oxidation state of metal cations.

The concentration dependence of the decomposition temperature of metastable ZnO—Li_n−2_MeO_n−1_ solid solutions (Me^n+^ = Sc^3+^, Ti^4+^, and Ta^5+^) at ambient pressure for all investigated systems is shown in Figure 11. It is easy to see that the thermal stability increases with the cation charge (n). Such behavior is apparently related to the difficulty of diffusion of multivalent ions in the cubic lattice.

It is known that there are several factors that determine the kinetic stability of metastable phases quenched from high pressure and/or high temperature. In the case of the pure cubic ZnO phase, it is the kinetic stabilization of the small grains of the high-pressure phase that prevents the appearance of wurtzite phase nuclei in a small volume [7]. High-entropy multicomponent solid solutions whose stabilization is determined by high values of configurational entropy have been described in the literature [44,45]. For example, an equimolar mixture of MgO, CoO, NiO, CuO, and ZnO oxides can be stabilized in the rock-salt structure, although two of these oxides (CuO and ZnO) do not form cubic polymorphs as individual oxides. The necessary condition for this type of stabilization is the same cation charge. In the case of the cubic solid solutions considered in the present work, these factors either do not work or their contribution can be estimated to be negligible [46].

In multiphase systems, one of the components can play the role of a “shell”, preventing the reverse phase transition of the metastable phase when the pressure is released. Such examples are described for the MgO-ZnO [47] and NaCl-ZnO [48] systems. Stability in such systems is provided either by the similar structure (MgO) or mechanical properties (NaCl) of the second component.

The octahedral environment preference factor [49] previously reported in [9,10,15] cannot be a determining factor in the case of ZnO solid solutions with Li_2_TiO_3_ and Li_3_TaO_4_. Although lithium tends to an octahedral environment, its radius (0.76 Å) is slightly larger than that of Zn^2+^ (0.74 Å) and the charge is half as low, so the influence of Li^+^ on the stability of the cubic solid solution cannot be significant. However, it should be noted that the strong ionicity of the Li-O bond (lithium has the lowest electronegativity value in the series of elements considered) is a favorable factor.

In the case of ternary systems, the stabilization of the metastable cubic ZnO could be explained by the presence of a multi-charged cation in the solid solution lattice, i.e., by the fact that a cation with a smaller radius but a higher charge (see Table 1) “compresses” the crystal lattice (the so-called “chemical pressure” effect), which is accompanied by a kinetic stabilization of the cubic structure in the form of solid solution. According to the deviation of the lattice parameters of solid solutions from Vegard’s law, the chemical pressure for titanium Ti^4+^ and tantalum Ta^5+^ in the ZnO-Li_2_TiO_3_ and ZnO-Li_3_TaO_4_ systems was estimated to be 3–5 GPa.

Thus, the metastable ZnO-Li_n−1_MeO_n−2_ solid solutions in Figure 11 are completely consistent with the charge of the Me^n+^ cation. Solid solutions with Sc^3+^, where the effect of chemical compression on the concentration dependence of the lattice parameter is not observed, exhibit the minimum thermal stability, and solid solutions with Ta^5+^ show the maximum.

We believe that some solid solutions with a rock-salt crystal structure, synthesized for the first time in the present work, may be of interest as promising Li-containing materials [50,51] or as materials with high dielectric characteristics [52,53].

The described approach of stabilizing metastable high-pressure phases under normal conditions by forming extended substitution solid solutions may also be promising for a number of other oxide and non-oxide (ZnS-LiGaS_2_, ZnS-Li_2_TiS_3_ and ZnS-Li_3_NbS_4_ [54,55,56]) systems whose high-pressure phases may have interesting functional properties. However, this hypothesis certainly requires experimental verification.

## 4. Conclusions

For the synthesized rock-salt ZnO—Li_n−2_MeO_n−1_ (Me = Sc^3+^, Ti^4+^, Ta^5+^) solid solutions, the limits of concentration (*x* up to 0.8) and thermal (up to 1000 K) stabilities were established. Coefficients of thermal expansion were determined for all the systems studied, showing either linear or quadratic dependence on temperature. The decomposition temperature of metastable solid solutions depends on the composition and nature of the multivalent cation, which allows us to define it as a chemical pressure effect.

## Figures and Tables

**Table 1:** Crystallographic data for Li_n−2_Me^n+^O_n−1_ complex oxides used as precursors for the synthesis of ZnO—Li_n−2_MeO_n−1_ solid solutions.

Precursor	Cation Radius (Å) *	Crystal Structure (Space Group)		References
LiScO_2_	Sc^3+^ (0.74)	*I*4_1_/*amd* (#141)		[19,20]
Li_2_TiO_3_	Ti^4+^ (0.60)	*C*2/*c* (#15) ^†^	*Fm*-3*m* (#225) ^‡^	[18,21,22,23,24,25]
Li_3_TaO_4_	Ta^5+^ (0.64)	*C*2/*c* (#15) ^§^	*P*2/*n* (#13) ^§^	[26,27,28,29]

* The ionic radii of Li^+^ (0.76 Å) and Zn^2+^ (0.74 Å) are given according to the Shannon scale [30] for coordination number 6. ^†^ At ambient pressure, monoclinic Li_2_TiO_3_ is thermodynamically stable in the 298–1430 K range [20]. ^‡^ At ambient pressure, rock-salt Li_2_TiO_3_ is thermodynamically stable between 1430 and 1820 K [21,22,23] and can be quenched to room temperature. The 300 K lattice parameter is 4.14281(5) Å [24] or 4.14276(1) Å [25], which is close to the lattice parameter of *rs*-ZnO (4.28 Å [5,8,9]). ^§^ At ambient pressure, the phase transition between the two monoclinic modifications of Li_3_TaO_4_ is observed at 1173 K [28)]. A metastable cubic (*Fm*-3*m*) Li_3_TaO_4_ has been reported to be kinetically stable below 1093 K [29,31]; its lattice parameter at room temperature is 4.2207(4) Å [31] or 4.214(5) Å [32].

**Table 2:** Parameters of Equation (2) describing the thermal expansion data of rock-salt solid solutions *x*ZnO—(1 − *x*)LiScO_2_ in the 300–900 K range (R^2^ = 99.9%).

Composition, *x*	V_0_ (Å^3^)	α_1_ × 10^5^ (K^−1^)	α_2_ × 10^8^ (K^−2^)
0.8	78.876(2)	3.70(4)	2.4(8)
0.7	79.115(4)	3.35(5)	3.2(1)

**Table 3:** Parameters of Equation (4) describing the thermal expansion data of rock-salt solid solutions *x*ZnO—(1 − *x*)Li_2_TiO_3_ in the 300–1000 K range (R^2^ > 99.8%).

Composition, *x*	V_0_ (Å^3^)	α × 10^5^ (K^−1^)
0.8	74.78(2)	5.42(6)
0.7	74.52(1)	5.75(3)
0.6	73.592(4)	6.16(2)
0.5	72.797(4)	6.39(1)
0.4	72.296(3)	6.20(1)
0.3	71.563(4)	6.39(1)
0.2	71.221(2)	6.47(1)
0.1	70.820(1)	6.42(1)
0.0 [25]	71.20(2)	6.52(4)

**Table 4:** Parameters of Equation (4) describing the thermal expansion data of rock-salt solid solutions *x*ZnO—(1 − *x*)Li_3_TaO_4_ in the 300–1000 K range (R^2^ > 99.8%).

Composition, *x*	V_0_ (Å^3^)	α × 10^5^ (K^−1^)
0.7	75.81(1)	6.79(3)
0.6	75.62(1)	6.81(2)
0.5	75.23(1)	6.67(1)

## Data Availability

The data presented in this study are available upon request.

## Supplementary Materials

The following supporting information can be downloaded at: https://www.mdpi.com/article/10.3390/ma16155336/s1.
