# Supplementary material for: Chemical Pressure Effect on the Stabilization of Rock-Salt ZnO—Lin−2MeOn−1 Solid Solutions Synthesized at High Pressure
Source: Materials (Basel). 2023 Jul 29;16(15):5336. doi: 10.3390/ma16155336 (PMC10419647; doi:10.3390/ma16155336)
Supplement: Supplementary file 1 [file materials-16-05336-s001.zip › materials-2507929-supplementary.pdf]

materials MDPI

Supplementary Materials

Chemical Pressure Effect on the Stabilization of Rock-Salt ZnO—$Li_{n-2}MeO_{n-1}$ Solid Solutions Synthesized at High Pressure

Petr S. Sokolov [1], Andrey N. Baranov [2] and Vladimir L. Solozhenko [1,*]

[1] LSPM–CNRS, Université Sorbonne Paris Nord, 93430 Villetaneuse, France
[2] Chemistry Department, Moscow State University, 119991 Moscow, Russia
* Correspondence: vladimir.solozhenko@univ-paris13.fr

Figure S1. Powder X-ray diffraction patterns of $Li_{n-2}MeO_{n-1}$ complex oxides (as-synthesized and quenched from 7.7 GPa and 1300 K).

Figure S2. Powder X-ray diffraction pattern of as-synthesized Li_2TiO_3.

Figure S3. Powder X-ray diffraction patterns of the rock-salt $(LiScO_2)_{0.2}(ZnO)_{0.8}$ (*a*), $(LiScO_2)_{0.3}(ZnO)_{0.7}$ (*b*), $(LiScO_2)_{0.5}(ZnO)_{0.5}$ (*c*), $(Li_2TiO_3)_{0.2}(ZnO)_{0.8}$ (*d*), $(Li_3TaO_4)_{0.2}(ZnO)_{0.8}$ (*e*) $(Li_3TaO_4)_{0.5}(ZnO)_{0.5}$ (*f*) solid solutions quenched from 7.7 GPa and 1300-1500 K.

Figure S4. Thermal expansion coefficient of rock-salt solid solutions $(Li_2TiO_3)_{1-x}(ZnO)_x$ depending on composition.

Figure S5. Unit cell volume of rock-salt Li_3TaO_4 *versus* temperature at ambient pressure.

Table S1. Parameters of Eq. (2) describing the thermal expansion data of rock-salt Li_3TaO_4 in the temperature range 300-1100 K.

Chemicals used for the synthesis of solid solutions in the systems ZnO – $Li_{n-2}MeO_{n-1}$ (n=3;4;5)

w-ZnO (Alfa Aesar) – ultra-pure, powder -325 mesh

Li_2CO_3 (Merck) – zur Analyse, powder min. 99%

Sc_2O_3 (Rhone-Progil S.A.) – 99.9%

TiO_2 (Prolabo) – pure

Ta_2O_5 (Merck) – pure

Phase purity of all starting reagents was confirmed by X-ray powder diffraction.

Chemical reactions for the preparation of $Li_{n-2}MeO_{n-1}$ *(n = 3; 4; 5)* complex oxides

$$Li_2CO_3 + Sc_2O_3 \rightarrow 2\ LiScO_2 + CO_2\uparrow \quad (S1)$$

$$Li_2CO_3 + TiO_2 \rightarrow Li_2TiO_3 + CO_2\uparrow \quad (S2)$$

$$3\ Li_2CO_3 + Ta_2O_5 \rightarrow 2\ Li_3TaO_4 + 3\ CO_2\uparrow \quad (S3)$$

The completeness of the reactions (S1, S2, S3) was checked by weight change and X-ray powder diffraction of the final complex oxides (Fig. S1, S2). All complex oxides were white fine powders.

Figure S1. Powder X-ray diffraction patterns of $Li_{n-2}MeO_{n-1}$ complex oxides (as-synthesized and quenched from 7.7 GPa and 1300 K).

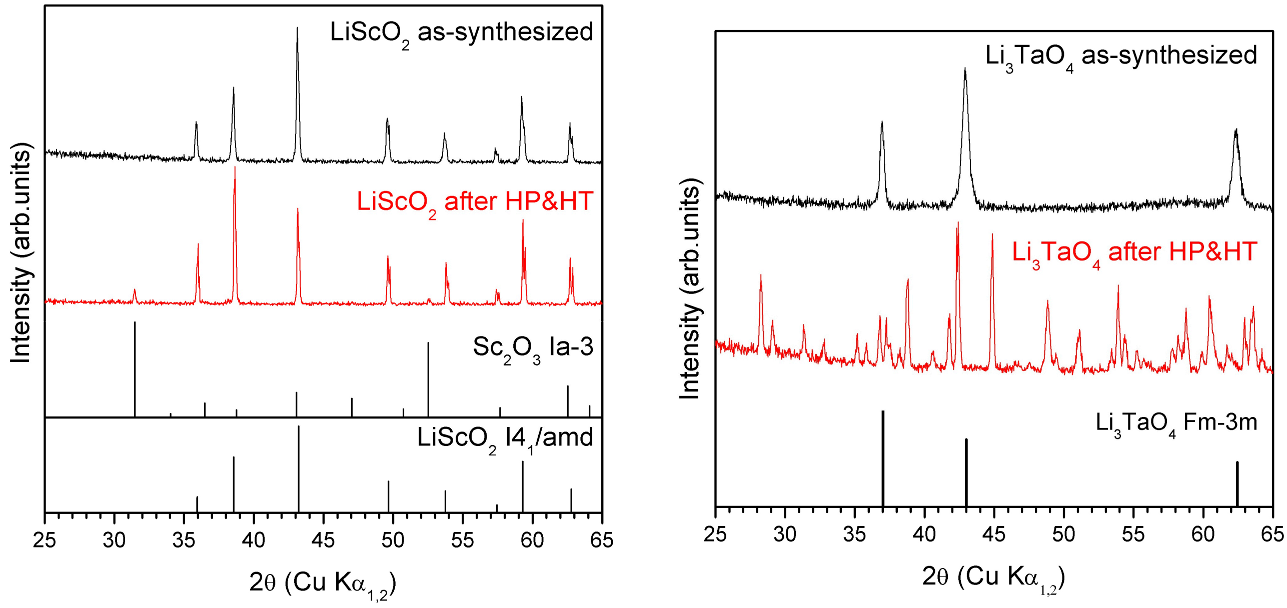

(**a**) (**b**)

Figure S2. Powder X-ray diffraction pattern of as-synthesized Li_2TiO_3.

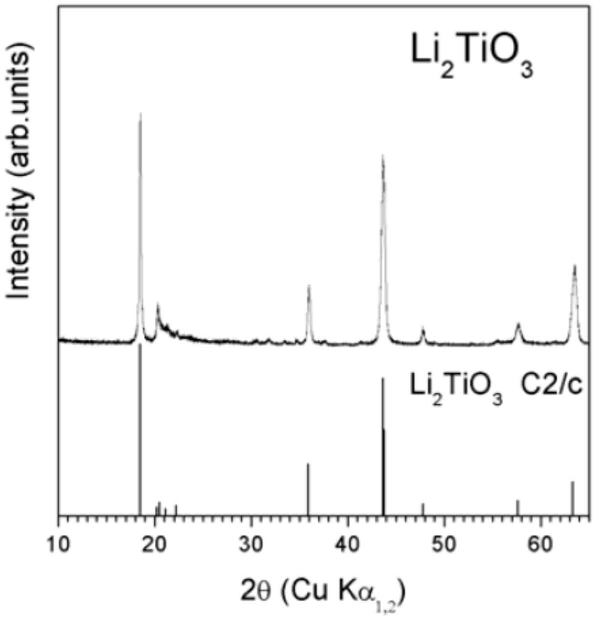

Figure S3. Powder X-ray diffraction patterns of the rock-salt $(LiScO_2)_{0.2}(ZnO)_{0.8}$ (*a*), $(LiScO_2)_{0.3}(ZnO)_{0.7}$ (*b*), $(LiScO_2)_{0.5}(ZnO)_{0.5}$ (*c*), $(Li_2TiO_3)_{0.2}(ZnO)_{0.8}$ (*d*), $(Li_3TaO_4)_{0.2}(ZnO)_{0.8}$ (*e*) and $(Li_3TaO_4)_{0.5}(ZnO)_{0.5}$ (*f*) solid solutions quenched from 7.7 GPa and 1300-1500 K. (*a*) λ=0.6876 Å; (*b*) λ=0.65148 Å; (*d*) λ=0.6841 Å; (*e*) and (*f*) λ=0.68805 Å.

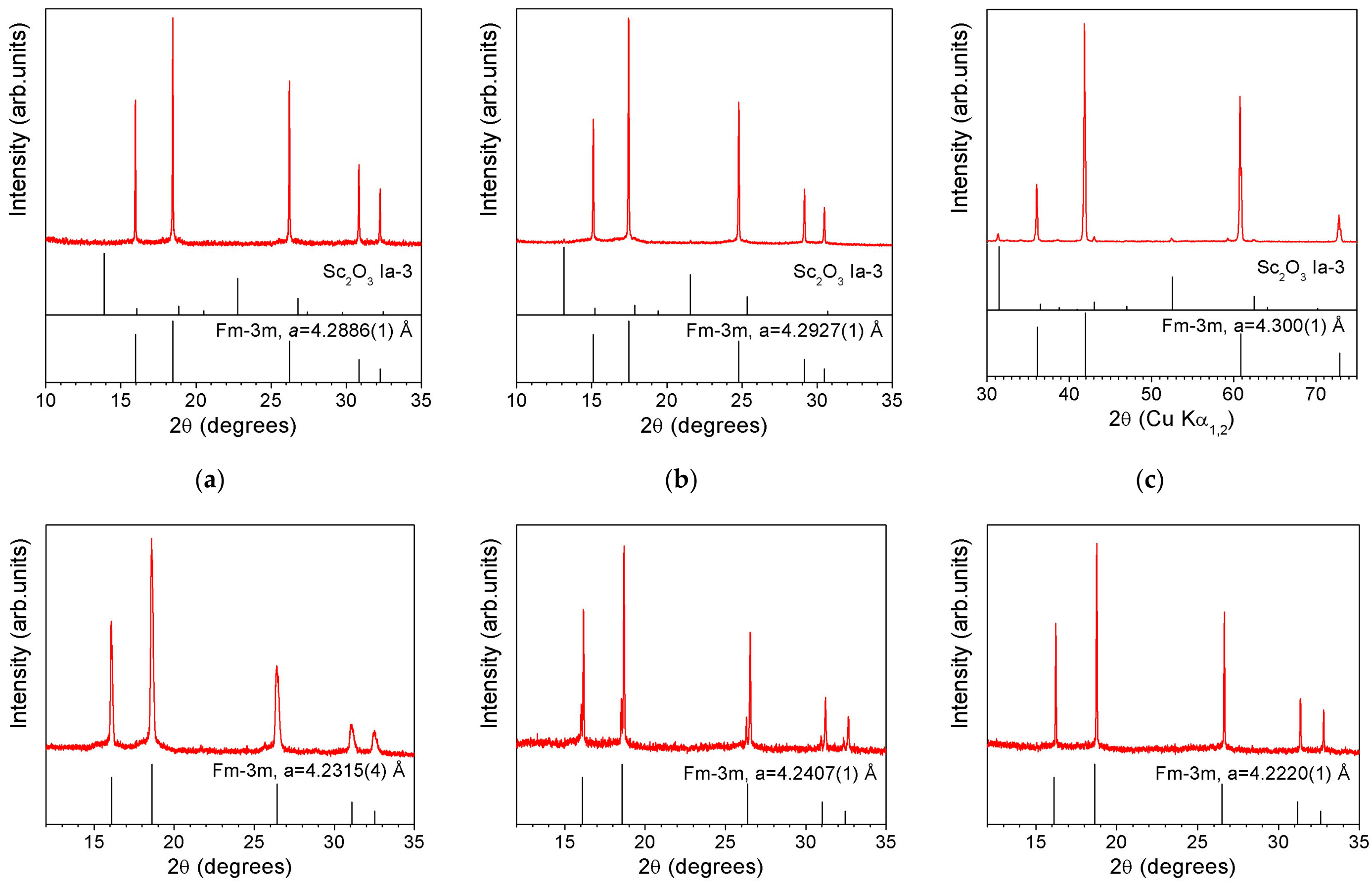

(**a**) (**b**) (**c**)

(**d**) (**e**) (**f**)

One can see that all case solid solutions have a cubic rock-salt structure and do not have impurity lines of *w* ZnO or lines of initial $Li_{n-2}MeO_{n-1}$ complex oxides.

Remark: In fact, *rs* $(Li_3TaO_4)_{0.5}(ZnO)_{0.5}$ and *rs* $(LiScO_2)_{0.5}(ZnO)_{0.5}$ solid solution can be presented as a new ternary compounds Li_3ZnTaO_5 and $LiZnScO_3$, respectively.

Figure S4. Thermal expansion coefficient of rock-salt solid solutions $(Li_2TiO_3)_{1-x}(ZnO)_x$ depending on composition. Red circle and solid square are literature data for pure *rs*-ZnO [10] and Li_2TiO_3 [26], respectively.

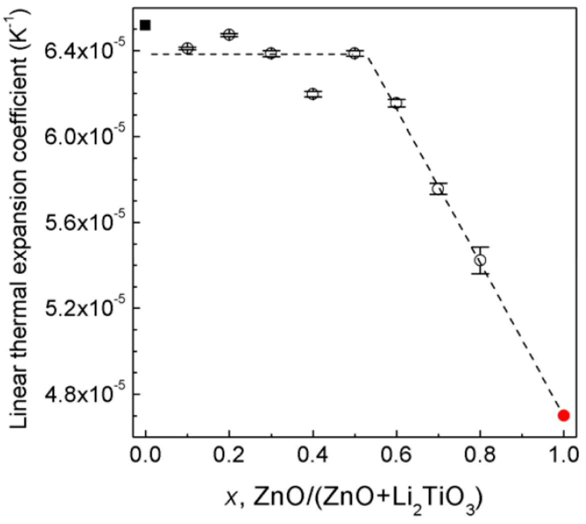

Figure S5. Unit cell volume of rock-salt Li_3TaO_4 *versus* temperature at ambient pressure. Error bars are smaller than the symbols. Dashed lines are least-squares fits (see Table S1 below).

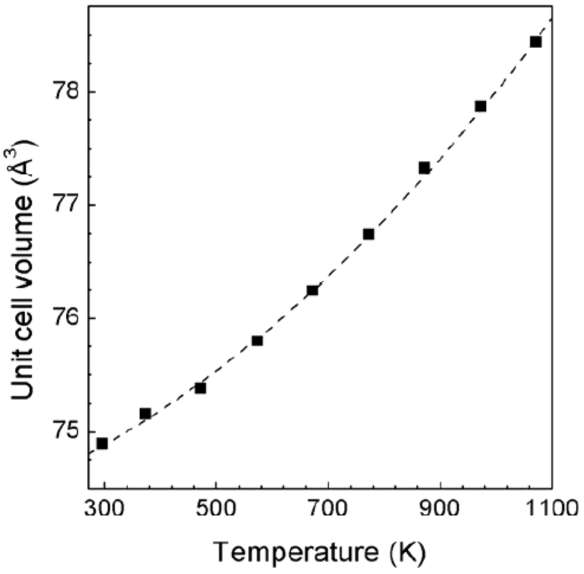

Table S1. Parameters of Eq. (2) describing the thermal expansion data of rock-salt Li_3TaO_4 in the temperature range 300-1100 K (R^2=99.9%).

V_0 (Å^3)	$\alpha_1 \times 10^5$ (K^{-1})	$\alpha_2 \times 10^8$ (K^{-2})
74.89(4)	3.61(3)	3.3(4)
